# HTLV-1 Tax Induces Formation of the Active Macromolecular IKK Complex by Generating Lys63- and Met1-Linked Hybrid Polyubiquitin Chains

**DOI:** 10.1371/journal.ppat.1006162

**Published:** 2017-01-19

**Authors:** Yuri Shibata, Fuminori Tokunaga, Eiji Goto, Ginga Komatsu, Jin Gohda, Yasushi Saeki, Keiji Tanaka, Hirotaka Takahashi, Tatsuya Sawasaki, Satoshi Inoue, Hiroyuki Oshiumi, Tsukasa Seya, Hiroyasu Nakano, Yuetsu Tanaka, Kazuhiro Iwai, Jun-ichiro Inoue

**Affiliations:** 1 Division of Cellular and Molecular Biology, Department of Cancer Biology, The Institute of Medical Science, The University of Tokyo, Tokyo, Japan; 2 Department of Pathobiochemistry, Graduate School of Medicine, Osaka City University, Osaka, Japan; 3 Research Center for Asian Infectious Diseases, The Institute of Medical Science, The University of Tokyo, Tokyo, Japan; 4 Laboratory of Protein Metabolism, Tokyo Metropolitan Institute of Medical Science, Tokyo, Japan; 5 Proteo-Science Center, Ehime University, Ehime, Japan; 6 Department of Anti-Aging Medicine, Graduate School of Medicine, The University of Tokyo, Tokyo, Japan; 7 Department of Microbiology and Immunology, Graduate School of Medicine, Hokkaido University, Sapporo, Japan; 8 Department of Biochemistry, Toho University School of Medicine, Tokyo, Japan; 9 Division of Immunology, Faculty of Medicine, University of the Ryukyus, Okinawa, Japan; 10 Department of Molecular and Cellular Physiology, Graduate School of Medicine, Kyoto University, Kyoto, Japan; Imperial College London, UNITED KINGDOM

## Abstract

The Tax protein of human T-cell leukemia virus type 1 (HTLV-1) is crucial for the development of adult T-cell leukemia (ATL), a highly malignant CD4^+^ T cell neoplasm. Among the multiple aberrant Tax-induced effects on cellular processes, persistent activation of transcription factor NF-κB, which is activated only transiently upon physiological stimulation, is essential for leukemogenesis. We and others have shown that Tax induces activation of the IκB kinase (IKK) complex, which is a critical step in NF-κB activation, by generating Lys63-linked polyubiquitin chains. However, the molecular mechanism underlying Tax-induced IKK activation is controversial and not fully understood. Here, we demonstrate that Tax recruits linear (Met1-linked) ubiquitin chain assembly complex (LUBAC) to the IKK complex and that Tax fails to induce IKK activation in cells that lack LUBAC activity. Mass spectrometric analyses revealed that both Lys63-linked and Met1-linked polyubiquitin chains are associated with the IKK complex. Furthermore, treatment of the IKK-associated polyubiquitin chains with Met1-linked-chain-specific deubiquitinase (OTULIN) resulted in the reduction of high molecular weight polyubiquitin chains and the generation of short Lys63-linked ubiquitin chains, indicating that Tax can induce the generation of Lys63- and Met1-linked hybrid polyubiquitin chains. We also demonstrate that Tax induces formation of the active macromolecular IKK complex and that the blocking of Tax-induced polyubiquitin chain synthesis inhibited formation of the macromolecular complex. Taken together, these results lead us to propose a novel model in which the hybrid-chain-dependent oligomerization of the IKK complex triggered by Tax leads to *trans*-autophosphorylation-mediated IKK activation.

## Author Summary

NF-κB is a key transcription factor that regulates many physiologically important cellular processes. However, persistent activation of NF-κB leads to chronic inflammation, autoimmunity and malignancy. Infection with the human retrovirus HTLV-1 causes adult T-cell leukemia, and HTLV-1 Tax-mediated persistent NF-κB activation is crucial for leukemogenesis. Therefore, a better understanding of the precise mechanism underlying aberrant NF-κB activation is essential to develop new therapeutic approaches. Ubiquitination is one of the major post-translational modifications that regulate various intracellular signaling pathways. We and others have shown that Tax activates NF-κB through activation of the IκB kinase (IKK) complex by generating Lys63-linked polyubiquitin chains. However, the molecular mechanism underlying Tax-induced IKK activation remains less well understood. Here, we demonstrate precisely how HTLV-1 Tax utilizes the ubiquitin system to activate the IKK complex. The IKK complex-associated Lys63/Met1-linked hybrid polyubiquitin chains are generated through the Tax-mediated recruitment of linear ubiquitin chain assembly complex (LUBAC) to the IKK complex. Furthermore, the hybrid chains are required for the Tax-induced formation of the active macromolecular IKK complex. Accordingly, we propose a novel model in which Tax triggers Lys63/Met1-linked hybrid-chain-dependent oligomerization of the IKK complex, leading to *trans*-autophosphorylation-mediated IKK activation.

## Introduction

Human T-cell leukemia virus type 1 (HTLV-1) is etiologically associated with adult T-cell leukemia (ATL), an aggressive and lethal malignancy of CD4^+^ T cells, and with HTLV-1-associated myelopathy/tropical spastic paraparesis (HAM/TSP) [1, 2]. The HTLV-1 provirus genome encodes a transactivator protein (Tax), which is crucial for viral gene expression and the onset and development of ATL together with another viral protein, HBZ [3–5]. Tax aberrantly activates host cell transcription factors, including nuclear factor-κB (NF-κB), cyclic AMP response element-binding protein (CREB) and serum responsive factor (SRF), thereby perturbing transcriptional networks in host cells [6]. Among these factors, accumulating evidence indicates that persistent activation of NF-κB by Tax is crucial for T cell transformation and ATL development [7–9].

NF-κB plays critical roles in immune responses, inflammation, bone metabolism, cell proliferation and survival [10]. NF-κB is composed of five Rel/NF-κB family members—p50/p105, p52/p100, RelA, RelB and c-Rel—which form various combinations of homo- and heterodimers. NF-κB is sequestered in the cytoplasm with inhibitory proteins of the NF-κB family (IκBs) or NF-κB precursors. Two distinct pathways lead to activation of NF-κB. The canonical pathway is activated by cytokines, such as tumor necrosis factor (TNF)-α and interleukin (IL)-1, whose stimulation leads to activation of the IκB kinase (IKK) complex, which is composed of the catalytic subunits IKKα and IKKβ and the regulatory subunit NEMO [11]. The IKK complex then induces phosphorylation and subsequent degradation of IκBα, which allows the p50/RelA heterodimer to translocate into the nucleus and activate target genes. In the noncanonical pathway, stimulation of CD40, receptor activator of NF-κB (RANK) or lymphotoxin-β receptor results in the activation of IKKα in a NIK-dependent but IKKβ- and NEMO-independent manner. IKKα then phosphorylates the C-terminal ankyrin repeats of p100, which forms heterodimers with RelB in the cytoplasm, leading to the proteasome-dependent selective degradation of the p100 C-terminal end to generate p52 [12]. The resulting p52/RelB heterodimer translocates into the nucleus and activates target genes. Tax is able to activate both canonical and noncanonical pathways, which are thought to be coordinately involved in leukemogenesis [13].

The importance of ubiquitination in the regulation of NF-κB activity is well established [14]. Ubiquitination is catalyzed by three enzymes in a stepwise fashion [15]. Ubiquitin-activating enzyme E1 forms a thioester linkage with ubiquitin, and the activated ubiquitin is then transferred to the E2 ubiquitin-conjugating enzyme. E2 acts as an escort for ubiquitin to the subsequent enzyme, E3 ligase, which binds to both E2 and the substrate and catalyzes the formation of an isopeptide bond between carboxylic acid at the C-terminal end of ubiquitin and the epsilon amine of the lysine residue in the substrate. After the addition of a single ubiquitin to the substrate, more ubiquitin can be repeatedly added to the previously conjugated molecule, thereby yielding a polyubiquitin chain. Ubiquitin itself contains seven lysines (K6, K11, K27, K29, K33, K48 and K63), each of which can participate in the formation of the ubiquitin chain, allowing seven linkage types [16]. In addition, ubiquitin can also be attached to the N-terminus of the proximal ubiquitin to generate a linear ubiquitin chain or Met1-linked ubiquitin chain (M1 chain) [17]. In the TNFR signaling pathway, several types of polyubiquitin chains cooperatively regulate IKK activation. Upon TNF-α stimulation, TNF receptor-1 recruits the adaptor TRADD, TRAF2/5 and cIAPs. cIAPs have E3 ligase activity and conjugate Lys11- and Lys63-linked ubiquitin chains (K11 and K63 chains) to RIP1 [18–20]. These polyubiquitin chains conjugated to RIP1 act as a scaffold for the formation of an active signaling complex containing transforming growth factor-β-activated kinase (TAK)-1, TAK1-binding (TAB) 2/3 and linear ubiquitin chain assembly complex (LUBAC) [21]. LUBAC is composed of HOIL-1L, HOIP and Sharpin [22, 23]. HOIP is the catalytic subunit while HOIL-1L and Sharpin are also required for the enzymatic activity of this complex. LUBAC conjugates M1 chains to NEMO [24], which may induce oligomer formation or a conformational change of NEMO to activate the IKK complex. Although previous studies have shown that Tax binds to NEMO and induces constitutive activation of the IKK complex in a K63-chain-dependent manner [25–28], the involvement of other types of polyubiquitin chains in Tax-induced IKK activation is still controversial [29].

In this study, we show that Tax induces generation of hybrids of K63 and M1 chains by recruiting LUBAC to the IKK complex, leading to the formation of the active macromolecular IKK complex. Thus, we propose a previously unidentified mechanism by which K63 and M1 chains cooperate in Tax-induced IKK activation.

## Results

### Tax-induced activation of the IKK complex requires the generation of K27, K63 and M1 chains

**Fig 1:**
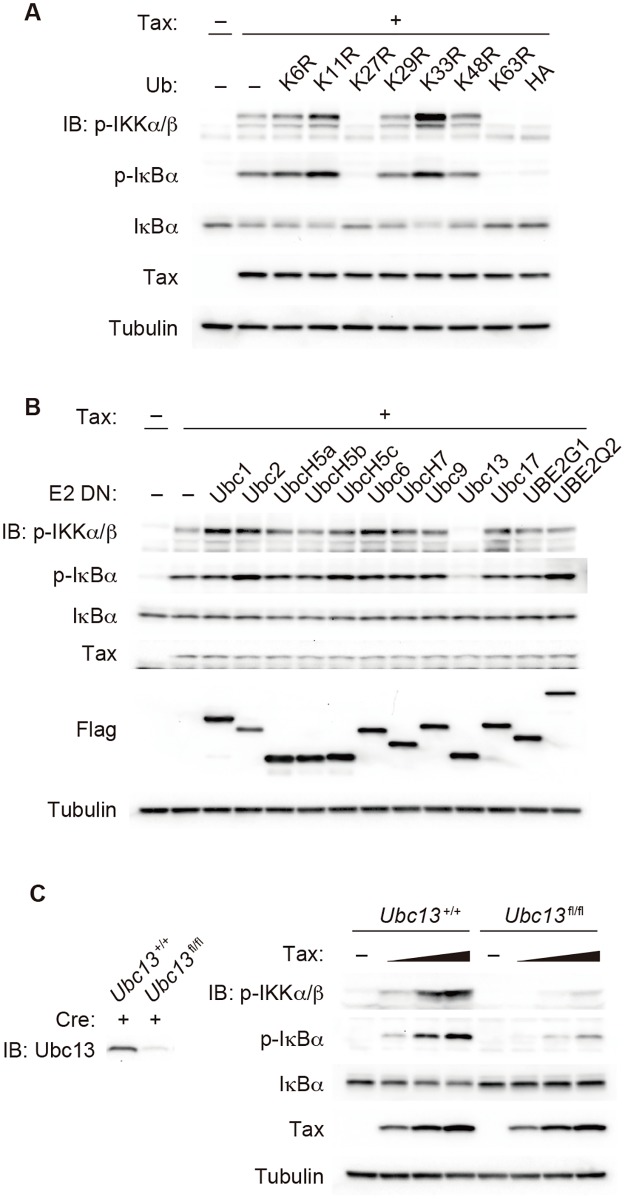
K27, K63 and M1 chains are involved in Tax-induced IKK activation. **(A)** Jurkat cytosolic extracts were incubated with recombinant His_6_-Tax and ATP (2 mM) in the presence of ubiquitin mutants or HA-ubiquitin (50 μM). The reaction mixtures were analyzed by immunoblotting with the indicated antibodies. **(B)** Cytosolic extracts were prepared from HEK293T cells expressing a series of dominant-negative mutants of the E2 enzyme and subjected to cell-free analyses. **(C)** Cytosolic extracts were prepared from WT MEFs or *Ubc13*^fl/fl^ MEFs expressing *Cre* and subjected to cell-free analyses with increasing amounts of Tax. The depicted results are representative of three independent experiments.

We previously established a cell-free assay to analyze Tax-induced IKK activation, in which the addition of recombinant Tax protein purified from *E*. *coli* into S-100 cytosolic extracts prepared from the Jurkat human T cell line, HEK293T cell line or mouse embryonic fibroblast (MEF) cells results in IKK activation [27]. To investigate which types of polyubiquitin linkages are required for Tax-induced IKK activation, we took advantage of a cell-free assay because the addition of dominant-negative (DN) ubiquitin mutants containing a single lysine-to-arginine substitution (K6R, K11R, K27R, K29R, K33R, K48R and K63R) or N-terminal HA-tagged ubiquitin results in linkage type-specific blockage of polyubiquitination. Immunoblots probed with anti-phospho-IKKα/β and phospho-IκBα antibodies revealed that the addition of K27R, K63R or HA-ubiquitin inhibited Tax-induced IKK activation (Fig 1A), suggesting that K27, K63 and M1 chains are required for IKK activation by Tax. Addition of K11R or K33R ubiquitin reproducibly enhanced Tax-induced IKK activation, probably because their addition could enhance the generation of K27, K63 or M1 chains. Note that phosphorylated IκBα is not degraded by proteasomes in a cell-free assay (S1 Fig), although the amount of IκBα was slightly reduced concomitantly with IκBα phosphorylation in some experiments in this paper. This could be due to the manufacturer-noted preferential binding of the anti-IκBα antibody used for immunoblotting to the non-phosphorylated form of IκBα. To identify the E2 ubiquitin-conjugating enzymes involved in Tax-induced IKK activation, a cell-free assay was performed using cytosolic extracts prepared from HEK293T cells expressing a series of E2 DN mutants, in which an active Cys residue was substituted with Ala. Expression of the Ubc13 DN mutant almost completely inhibited IKK activation, whereas other E2 DN mutants did not (Fig 1B). A cell-free assay using the extract from *Ubc13*^−/−^ MEFs further confirmed that Ubc13 is required for Tax-induced IKK activation (Fig 1C), which is consistent with previous reports based on experiments using intact *Ubc13*^−/−^ MEFs and a cell-free assay using the extract from Ubc13-knockdown cells [28, 30]. Low-level Tax-induced phosphorylation of IκBα was observed in *Ubc13*^−/−^ MEFs, which could be due to residual Ubc13 attributable to incomplete gene disruption by the Cre/loxP system. The lack of candidate E2 enzymes other than Ubc13 suggests that several E2 enzymes may redundantly generate K27 and M1 chains or that E2 enzymes not tested here could be involved. Because it has been reported that RNF8, as an E3 ubiquitin ligase, is partially involved in the Tax-induced generation of K63 chains [28], we checked whether other E3 enzymes capable of generating K63 chains are involved [18, 31–33]. Cytosolic extracts were prepared from cIAP1/cIAP2-deficient (*Birc2*^−/−^/*Birc3*^−/−^), TRAF2/TRAF5-deficient (*Traf2*^−/−^/*Traf5*^−/−^), TRIM25-deficient (*Trim25*^−/−^) and Riplet-deficient (*Rnf135*^−/−^) MEFs [33–36] and were subjected to a cell-free assay. None of the extracts derived from the mutant cells showed reduced IKK activation (S2 Fig), suggesting that these E3 enzymes are not involved in Tax-induced IKK activation. In addition, TRAF6, another E3 enzyme, has been shown to be dispensable in Tax-induced IKK activation but instead can work together with Ubc13 to generate K63 chains for cytokine-induced IKK activation [27, 37]. We then hypothesized that Tax itself may be an E3 ligase as recently proposed [29], since Tax contains a putative zinc finger domain at its N-terminus (S3A Fig) [38] and the zinc finger domain may act as a catalytic domain of E3 ligase as previously shown in the zinc finger of A20 [39]. Some zinc finger mutants of Tax failed to activate the IKK complex and NF-κB (S3B and S3C Fig), indicating that the zinc finger of Tax is crucial for IKK activation. Although recombinant Tax purified from either *E*. *coli* or Sf9 cells can efficiently activate IKK (S3D Fig), neither of them induced polyubiquitination in the presence of E2 enzymes including UbcH5c, UbcH7 and Ubc13/Uev1A under conditions that allow TRAF6 to generate polyubiquitin chains together with Ubc13/Uev1A (S3E Fig). These results strongly suggest that Tax itself does not possess E3 ligase activity.

### Tax requires LUBAC to induce IKK activation

**Fig 2:**
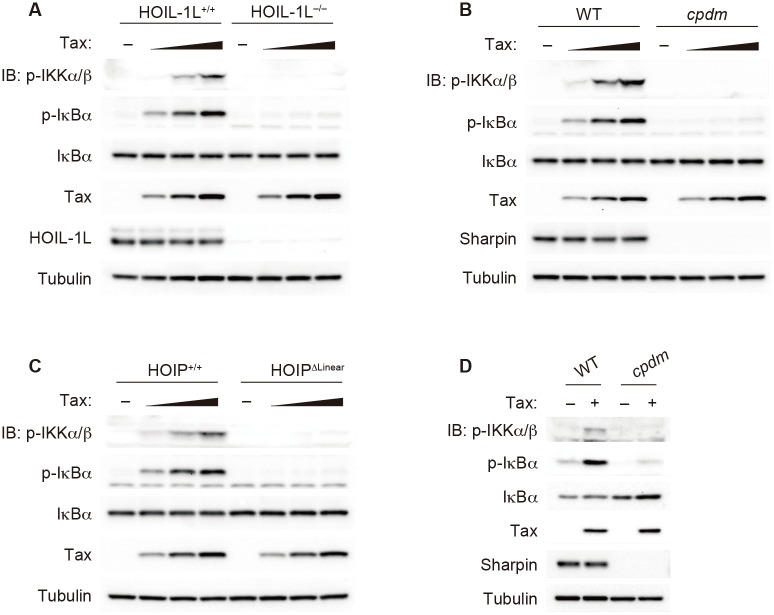
LUBAC is required for Tax-induced IKK activation. **(A-C)** Cytosolic extracts were prepared from HOIL-1L-deficient **(A)**, Sharpin-deficient **(B)**, HOIP^Δlinear^ **(C)** and corresponding WT MEFs and subjected to cell-free analyses. (**D)** Sharpin-deficient and corresponding WT MEFs were mock-infected or were transduced with retroviruses expressing Tax. The MEFs were treated with MG132 (10 μM) for 1 h, and cell lysates were subjected to immunoblotting with the indicated antibodies. The depicted results are representative of three independent experiments.

To further confirm the requirement for M1 chains, cytosolic extracts derived from MEFs that lack each component of LUBAC (the only known E3 ligase complex that catalyzes M1 chain generation) were tested. Tax failed to induce IKK activation when cytosolic extracts from HOIL-1L-deficient (*Rbck1*^−/−^) MEFs, Sharpin-deficient (*cpdm*) MEFs or MEFs in which the RING-IBR-RING region (the catalytic center) of HOIP was ablated (HOIP^Δlinear^) were used (Fig 2A–2C) [40]. To confirm the requirement for LUBAC for Tax-induced IKK activation in intact cells, Sharpin-deficient MEFs were infected with a Tax-expressing retrovirus, and subsequent phosphorylation of IKK and IκBα was detected by immunoblotting. Tax-induced IKK activation was significantly reduced in cells that lack LUBAC activity (Fig 2D). Taken together, these results clearly indicate that LUBAC is crucial for Tax-induced IKK activation.

### Tax recruits LUBAC to the IKK complex

**Fig 3:**
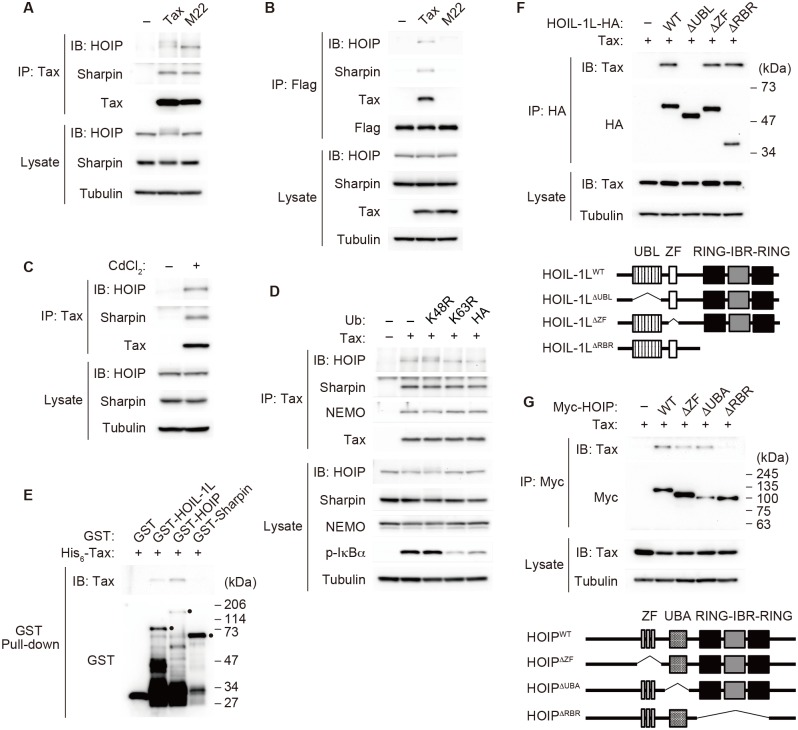
Tax recruits LUBAC to the IKK complex. **(A)** Jurkat cytosolic extracts were incubated with recombinant His_6_-Tax or His_6_-M22 in the presence of ATP (2 mM). The reaction mixtures were subjected to immunoprecipitation with an anti-Tax antibody, followed by immunoblotting with the indicated antibodies. **(B)** Cytosolic extracts prepared from Jurkat cells expressing Flag-tagged NEMO were incubated with recombinant His_6_-Tax or His_6_-M22 in the presence of ATP (2 mM). The reaction mixtures were subjected to immunoprecipitation with an anti-Flag antibody, followed by immunoblotting with the indicated antibodies. **(C)** JPX-9 cells were untreated or treated with CdCl_2_ (20 μM) for 18 h. Cell lysates were subjected to immunoprecipitation with an anti-Tax antibody, followed by immunoblotting with the indicated antibodies. **(D)** Jurkat cytosolic extracts were incubated with recombinant His_6_-Tax and ATP (2 mM) in the presence of ubiquitin mutants or HA-ubiquitin (50 μM). The reaction mixtures were subjected to immunoprecipitation with an anti-Tax antibody, followed by immunoblotting with the indicated antibodies. **(E)** Recombinant His_6_-Tax was incubated with glutathione sepharose-bound recombinant GST, GST-HOIL-1L, GST-HOIP or GST-Sharpin. His_6_-Tax bound to GST-tagged protein was analyzed by immunoblotting with an anti-Tax antibody. Dots denote full-length GST fusion proteins. **(F)** HEK293T cells were transfected with expression plasmids encoding HOIL-1L-HA or various C-terminal HA-tagged HOIL-1L mutants together with a Tax expression plasmid. After 48 h, cell lysates were prepared and subjected to immunoprecipitation with an anti-HA antibody, followed by immunoblotting with an anti-Tax antibody (upper). A schematic representation of the various mutants of HOIL-1L (lower). **(G)** HEK293T cells were transfected with expression plasmids encoding Myc-HOIP or various N-terminal Myc-tagged HOIP mutants together with a Tax expression plasmid. After 48 h, cell lysates were prepared and subjected to immunoprecipitation with an anti-Myc antibody, followed by immunoblotting with an anti-Tax antibody (upper). A schematic representation of the various mutants of HOIP (lower). The depicted results are representative of three independent experiments.

To understand how LUBAC is involved in Tax-induced IKK activation, we first investigated whether LUBAC binds to Tax. When Tax was immunoprecipitated with an anti-Tax antibody after incubation with Jurkat cytosolic extracts, HOIP and Sharpin were co-immunoprecipitated with Tax (Fig 3A), indicating that Tax interacts with LUBAC. In addition, the Tax mutant M22, which is incapable of activating NF-κB due to a lack of binding ability to NEMO [30], also bound to HOIP and Sharpin (Fig 3A), indicating that the binding of Tax to LUBAC is not mediated by the IKK complex. This result led us to hypothesize that Tax acts as an adaptor in the formation of a multi-protein complex composed of LUBAC, Tax and the IKK complex. To test this hypothesis, the IKK complex was immunoprecipitated with an anti-Flag antibody from cytosolic extracts of Jurkat cells expressing Flag-NEMO. HOIP and Sharpin were recruited to the IKK complex in the presence of Tax, but M22 failed to recruit LUBAC to the IKK complex (Fig 3B), indicating that Tax functions as an adaptor to recruit LUBAC to the IKK complex. We then sought to determine whether Tax also acts as a bridge between LUBAC and the IKK complex in an intact Jurkat human T cell line. JPX-9, a Jurkat-derived cell line in which Tax expression is induced by Cd^2+^ treatment [41], was first cultured in the presence or absence of Cd^2+^, and cell lysates were subjected to immunoprecipitation using an anti-Tax antibody. HOIP and Sharpin were included in the immunoprecipitates only when Tax was induced (Fig 3C). These results indicate that Tax associates with LUBAC in intact T cells. Interestingly, the slower-migrating form of HOIP was observed only when the IKK complex was activated by Tax (Fig 3A). This band shift was due to the phosphorylation of HOIP because Phos-tag SDS-PAGE analysis identified slower-migrating bands (S4A Fig). Treatment of lysates with the IKKβ inhibitor TPCA-1 resulted in the disappearance of the slower-migrating bands in a dose-dependent manner (S4B Fig), suggesting that IKKβ phosphorylates HOIP during Tax-induced IKK activation. The significance of HOIP phosphorylation in IKK activation remains to be elucidated.

Given that HOIP binds to K63 chains but not M1 chains [42], we hypothesized that K63 chains are required for the binding of LUBAC to the IKK complex. To test this possibility, we first investigated whether the addition of DN ubiquitin mutants would inhibit the Tax-mediated binding of LUBAC to the IKK complex. The addition of K63R or HA-tagged ubiquitin inhibited Tax-induced IKK activation (Fig 1A), whereas the Tax-mediated binding of LUBAC to the IKK complex was not affected (Fig 3D). These results indicate that K63 and M1 chains are not required for the binding of Tax to LUBAC and the IKK complex. To determine which components of LUBAC bind to Tax and also whether the binding is direct, an *in vitro* binding assay was performed using purified recombinant proteins. Purified GST-HOIL-1L, GST-Sharpin or GST-HOIP was incubated with His_6_-Tax and subjected to GST pull-down assay. GST-HOIL-1L and GST-HOIP bound to His_6_-Tax, whereas GST-Sharpin did not (Fig 3E), indicating that HOIL-1L and HOIP directly bind to Tax. To elucidate the molecular basis of the binding of HOIL-1L or HOIP to Tax, a series of deletion mutants of HOIL-1L and those of HOIP were tested by co-immunoprecipitation assay. HOIL-1L ΔUBL and HOIP ΔRBR failed to bind to Tax, whereas the other mutants of HOIL-1L and HOIP proteins bound to Tax as efficiently as the full-length protein (Fig 3F and 3G). These results indicate that HOIL-1L and HOIP interact directly with Tax through their UBL and RBR domains, respectively.

### Tax-induced generation of K63/M1-linked hybrid chains associated with IKK complex is required for IKK activation

**Fig 4:**
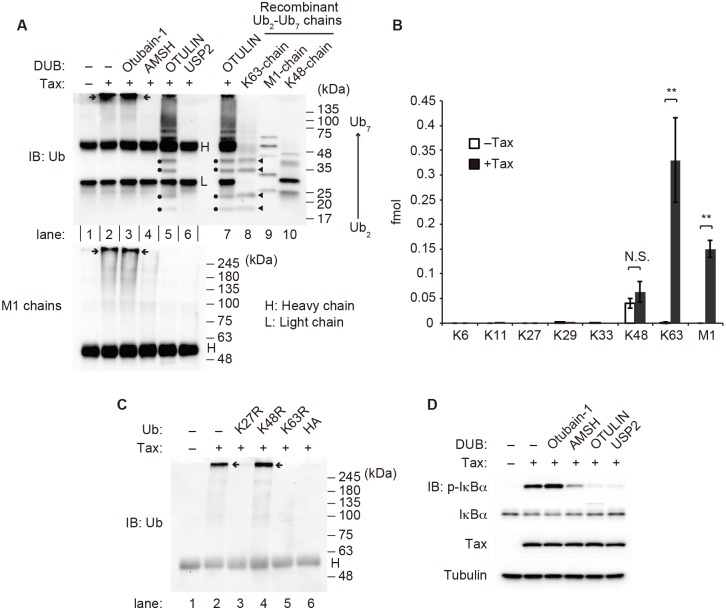
Tax-induced generation of IKK complex-associated K63/M1-linked hybrid chains is required for IKK activation. **(A)** Jurkat cytosolic extracts were incubated with recombinant His_6_-Tax and ATP (2 mM), and the reaction mixtures were subjected to immunoprecipitation with an anti-NEMO antibody. The immunoprecipitates were left untreated or were treated with recombinant Otubain-1, AMSH, OTULIN or USP2 and analyzed by immunoblotting with anti-Ub (upper) and anti-M1 chain-specific (lower) antibodies. **(B)** Jurkat cytosolic extracts were incubated with or without recombinant His_6_-Tax and ATP (2 mM), and the reaction mixtures were subjected to immunoprecipitation with an anti-NEMO antibody. The immunoprecipitates were analyzed via the ubiquitin-AQUA method. The results are given as the mean ± SD (*n* = 3). **(C)** Cell-free reactions were performed in the presence of ubiquitin mutants or HA-ubiquitin (50 μM). The reaction mixtures were subjected to immunoprecipitation with an anti-NEMO antibody, followed by immunoblotting with an anti-Ub antibody. **(D)** Cell-free reactions were performed in the presence of various DUBs. The depicted results are representative of three independent experiments.

To determine how the Tax-induced generation of polyubiquitin chains leads to IKK activation, Jurkat cytosolic extracts were incubated in the absence or presence of recombinant Tax, and the reaction mixtures were then subjected to immunoprecipitation with an anti-NEMO antibody. The resulting immunoprecipitates were immunoblotted with either an anti-ubiquitin (Ub) antibody that can recognize monoubiquitin and any type of polyubiquitin linkages or an anti-M1 chain-specific antibody. Both antibodies clearly detected smeared bands only when cytosolic extracts were incubated with Tax (Fig 4A, lane 2), indicating that M1 chains were associated with the IKK complex in a Tax-dependent manner. To further characterize the IKK complex-associated ubiquitin chains, the immunocomplexes precipitated with an anti-NEMO antibody were then treated with the following chain type-specific deubiquitinases (DUBs): Otubain-1 for K48 chains [43, 44], associated molecule with the SH3 domain of STAM (AMSH) for K63 chains [42, 45], OTULIN for M1 chains [46], and ubiquitin-specific protease 2 (USP2) for any type of polyubiquitin chain [47]. Otubain-1 treatment did not affect the smears detected by the anti-Ub or anti-M1 chain antibody (Fig 4A, lane 3 upper and lower), indicating that K48 chains were nearly nonexistent in the complex. In contrast, AMSH treatment almost completely abolished the smears detected by the anti-Ub antibody (Fig 4A, lane 4 upper), and OTULIN treatment completely abolished the smears detected by the anti-M1 chain antibody (Fig 4A, lane 5 lower). These results indicated that both K63 and M1 chains were associated with the IKK complex. These results were further supported by the quantification of different ubiquitin chain types associated with the IKK complex via the ubiquitin-AQUA method using mass spectrometry [48]. While residual amounts of K48 chains were detected irrespective of the presence of Tax, an approximately 2:1 ratio of K63 to M1 chain linkages was significantly associated with the IKK complex only when cytosolic extracts were incubated with Tax (Fig 4B).

Interestingly, when an immunoblot of the AMSH-treated IKK complex was probed with the anti-M1 chain antibody, extremely high-molecular-weight ubiquitin-containing complexes (EHUCs), which remained at the top of separating gels (arrows in Fig 4A and 4C), were degraded (Fig 4A, lane 4 lower), indicating that EHUCs are polyubiquitin chains that include both K63 and M1 linkages in a single chain. This notion was also supported by experiments showing that Tax failed to generate EHUCs when either the generation of K63 chains or that of M1 chains was blocked (Fig 4C, lanes 5 and 6). Furthermore, when an immunoblot of the OTULIN-treated IKK complex was probed with the anti-Ub antibody, the abundance of EHUCs was found to be significantly reduced while ladders between 17 and 75 kDa appeared (Fig 4A, lanes 5 and 7 upper, note that the same sample was applied in lanes 5 and 7). Importantly, among these ladders, four bands (dots in Fig 4A, lanes 5 and 7 upper) migrated almost identically to the bands corresponding to trimer (Ub_3_), tetramer (Ub_4_), pentamer (Ub_5_) and hexamer (Ub_6_) of recombinant K63 chains (arrowheads in Fig 4A, lane 8 upper). These results clearly indicate that K63/M1-linked hybrid chains are associated with the IKK complex activated by Tax.

To address whether these IKK complex-associated polyubiquitin chains are required for Tax-induced IKK activation, cell-free assays were performed in the presence of various DUBs. AMSH, OTULIN and USP2, but not Otubain-1, inhibited the phosphorylation of IκBα induced by Tax (Fig 4D), indicating that generation of both K63 and M1 chains is essential for Tax-induced IKK activation. Therefore, generation of IKK complex-associated K63/M1-linked hybrid chains is likely to be essential for Tax-induced IKK activation.

### K63/M1-linked hybrid chains are required for the formation of the macromolecular complex of IKK

**Fig 5:**
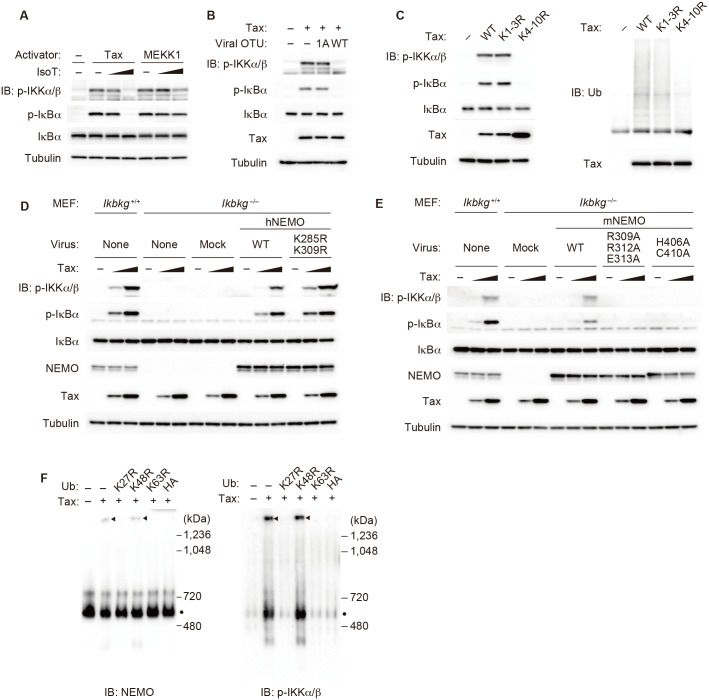
Tax induces formation of the macromolecular active IKK complex. **(A)** Jurkat cytosolic extracts were incubated with recombinant His_6_-Tax or His_6_-MEKK1 together with ATP (2 mM) in the presence or absence of IsoT. **(B)** Cell-free reactions were performed in the presence of WT viral OTU or its catalytic inactive mutant 1A. **(C)** Jurkat cytosolic extracts were incubated with recombinant His_6_-Tax or its mutants together with ATP (2 mM). The reaction mixtures were subjected to immunoprecipitation with an anti-Tax antibody, followed by immunoblotting with an anti-Ub antibody. **(D)** Cytosolic extracts were prepared from NEMO-deficient MEFs reconstituted with human NEMO or its mutant and were subjected to cell-free analyses. **(E)** Cytosolic extracts were prepared from NEMO-deficient MEFs reconstituted with mouse NEMO or its mutants and were subjected to cell-free analyses. **(F)** Cell-free reactions were performed, and the reaction mixtures were subjected to Blue native-PAGE, followed by immunoblotting with anti-NEMO (left) and anti-p-IKKα/β (right) antibodies. The depicted results are representative of three independent experiments.

In the cytokine-induced NF-κB signaling pathway, IKK activation requires the formation of unanchored K63 chains or NEMO-conjugated M1 chains [24, 49]. To understand the roles of unanchored and substrate-conjugated (anchored) chains in Tax-induced IKK activation, cell-free assays were performed in the presence of Isopeptidase T (IsoT), a DUB specific for unanchored chains, or the OTU domain of the L protein of Crimean Congo hemorrhagic fever virus (viral OTU), a DUB specific for substrate-anchored chains. IsoT inhibited the Tax-induced phosphorylation of IKK and IκBα but not their polyubiquitination-independent phosphorylation by MEKK1 (Fig 5A). In addition, viral OTU, but not its catalytic inactive mutant (1A), inhibited the Tax-induced phosphorylation of IKK and IκBα (Fig 5B). These results indicate that both unanchored and substrate-anchored polyubiquitin chains are required for Tax-induced IKK activation. Several studies have shown that ubiquitination of Tax is required for IKK activation [50–52]. Among ten lysine residues present in Tax, ubiquitination of the C-terminal seven lysines (K4 to K10) are required for Tax-induced IKK activation in intact cells [50]. To examine whether Tax requires similar ubiquitination for IKK activation in our cell-free system, recombinant Tax mutants containing lysine-to-arginine mutations at the three N-terminal lysines (K1-3R) or at the seven C-terminal lysines (K4-10R) were generated. Both Tax-WT and the K1-3R mutant induced IKK activation equally well, whereas the K4-10R mutant did not (Fig 5C left). In addition, the K4-10R mutation significantly reduced Tax ubiquitination (Fig 5C right). These results strongly suggest that the polyubiquitin chains conjugated to Tax belong to the class of substrate-anchored polyubiquitin chains required for Tax-induced IKK activation.

Because LUBAC induces polyubiquitination at K285 and K309 of NEMO in cytokine-induced IKK activation [24], we next investigated whether these lysine residues are required for Tax-induced IKK activation. Human NEMO-WT or its mutant (K285R/K309R) was introduced into NEMO-deficient (*Ikbkg*^−/−^) MEFs, and cell-free assays were performed. Tax induced phosphorylation of IKK and IκBα equally well in NEMO-WT- and NEMO (K285R/K309R)-expressing cytosolic extracts (Fig 5D), indicating that polyubiquitination at K285 and K309 of NEMO is dispensable for Tax-induced IKK activation. It has been reported that the binding of NEMO to K63 and M1 chains is required for cytokine-induced IKK activation. NEMO binds to K63 chains through the C-terminal NZF domain and to M1 chains through the UBAN domain [53, 54]. To determine the requirement for the binding ability of NEMO to K63 or M1 chains, cytosolic extracts were prepared from NEMO-deficient MEFs expressing mouse NEMO-WT, its mutant (R309A/R312A/E313A) lacking the ability to bind to M1 chains or another NEMO mutant (H406A/C410A) lacking the ability to bind to K63 chains. Tax failed to induce IKK activation in cytosolic extracts expressing the NEMO (R309A/R312A/E313A) or NEMO (H406A/C410A) mutant (Fig 5E), indicating that the binding ability of NEMO to both K63 and M1 chains is required for Tax-induced IKK activation.

Because the ability of NEMO to bind to both K63 and M1 chains and the Tax-induced generation of the IKK complex-associated K63/M1-linked hybrid chains are required for the activation of IKK by Tax, we hypothesized that the macromolecular complex of IKK may be formed through multivalent interactions between polyubiquitin chains and NEMO, facilitating *trans*-autophosphorylation between the IKK complexes, thereby inducing IKK activation. To test this possibility, we performed cell-free assays in the presence or absence of DN ubiquitin mutants, and the reaction mixtures were subjected to Blue native-PAGE, which can be used to determine the size and composition of native protein complexes [55], followed by immunoblotting. Probing the immunoblots with an anti-NEMO antibody revealed that the NEMO-containing macromolecular complex (arrowheads in Fig 5F left) was formed only in the presence of Tax in addition to the regular complex of approximately 600 kDa (dots in Fig 5F), which is observed as an inactive IKK complex in the absence of Tax. Interestingly, the formation of the macromolecular complex was abrogated when the extract was incubated with DN ubiquitin mutants (K27R, K63R, or HA-Ub) that also inhibit Tax-induced IKK activation (Fig 5F left). Furthermore, probing the immunoblots with an anti-p-IKKα/β antibody revealed that activated IKK was observed only when the macromolecular complex was formed and that activated IKK was included in the macromolecular complex (Fig 5F right). These results strongly suggest that polyubiquitination-dependent formation of the macromolecular IKK complex triggers IKK activation.

### LUBAC associates with Tax and is involved in NF-κB activation in HTLV-1-infected cells

**Fig 6:**
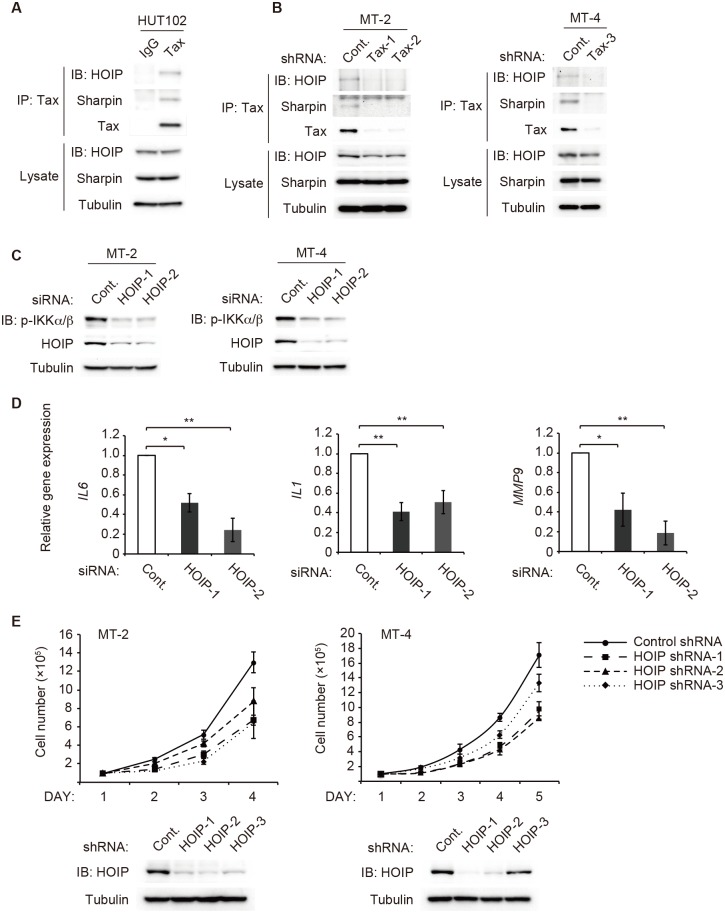
LUBAC associates with Tax and is involved in NF-κB activation leading to target gene expression and cell proliferation in HTLV-1-infected cells. **(A)** Cell lysates from HUT102 cells were subjected to immunoprecipitation with an anti-Tax antibody or a control antibody, followed by immunoblotting with the indicated antibodies. **(B)** MT-2 (left) or MT-4 (right) cells were infected with control or Tax shRNA expression lentivirus vector. Cell lysates were subjected to immunoprecipitation with an anti-Tax antibody, followed by immunoblotting with the indicated antibodies. **(C)** MT-2 (left) or MT-4 (right) cells were transfected with control or HOIP siRNA. Cell lysates were subjected to immunoblotting with an anti-pIKKα/β antibody. **(D)** MT-4 cells were transfected with control or HOIP siRNA as described in **(C)**. The expression levels of *IL6* (left), *IL1* (middle) and *MMP9* (right) were measured by quantitative real-time RT-PCR. The relative expression was calculated by dividing each expression value by that of a control siRNA treatment. **(E)** MT-2 (left) or MT-4 (right) cells were infected with HOIP shRNA expression lentivirus vector. The cells were seeded at 1.0×10^5^ cells/well, and cell viability was assessed by trypan blue exclusion assay (upper). The whole cell lysates were subjected to immunoblotting with an anti-HOIP antibody to confirm the knockdown of HOIP (lower). The results shown in **(D)** and **(E)** are given as the mean ± SD (*n* = 3). The depicted results are representative of three independent experiments.

To investigate the physiological significance of LUBAC in HTLV-1-infected cells, we first checked the interaction between Tax and LUBAC. Lysates prepared from the HTLV-1-infected human T cell line HUT102, which is derived from a mycosis fungoides patient [56], were subjected to immunoprecipitation using an anti-Tax or a control antibody. HOIP and Sharpin were precipitated only when Tax was precipitated by the anti-Tax antibody (Fig 6A). To further confirm the physiological interaction between Tax and LUBAC, MT-2 or MT-4 cell lines, T cell lines transformed by co-culture with HTLV-1-producing ATL cells [57, 58], were used. HOIP and Sharpin were co-precipitated with Tax using an anti-Tax antibody. However, neither HOIP nor Sharpin were precipitated when Tax was knockdown (Fig 6B). These results indicate that Tax interacts with endogenous LUBAC in three distinct HTLV-1-infected cell lines. To understand whether LUBAC is involved in IKK activation in HTLV-1-infected cells, effect of HOIP knockdown on IKK activation was analyzed. Immunoblotting with an anti-pIKKα/β antibody revealed that HOIP knockdown blocked IKK activation in MT-2 and MT-4 cells (Fig 6C). Consistently, expression levels of the NF-κB target genes were notably reduced in HOIP-knockdown MT-4 cells (Fig 6D). Moreover, HOIP knockdown significantly suppressed cell proliferation (Fig 6E). Taken together, these results show that LUBAC-mediated M1 chain formation is required for NF-κB activation leading to target gene expression and cell proliferation in HTLV-1-infected cells.

## Discussion

**Fig 7:**
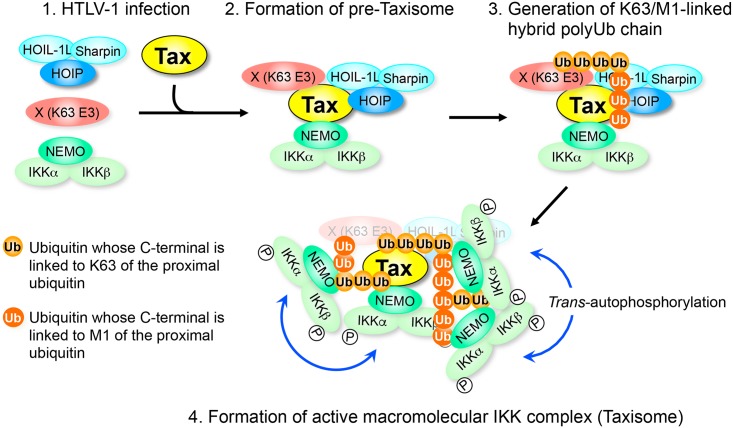
A model illustrating HTLV-1 Tax-induced IKK activation. Upon HTLV-1 infection, the Tax protein is translated from doubly spliced viral mRNA expressed from the provirus. Tax is able to bind to NEMO in the IKK complex and to HOIP and HOIL-1L of LUBAC, thereby forming the LUBAC/Tax/IKK complex, which further binds to the Ubc13/Uev1A E2 complex and unidentified K63 E3 enzymes (X) to generate an inactive “pre-Taxisome”. The K63/M1-linked hybrid polyubiquitin chains generated by LUBAC and X (K63 E3) interact with NEMO through its M1 chain-interacting UBAN domain and K63 chain-interacting NZF domain. This multivalent interaction between NEMO proteins and the hybrid polyubiquitin chains results in the oligomerization of the pre-Taxisome to form the macromolecular Taxisome, which allows close interactions between the IKK complexes, leading to the *trans*-autophosphorylation-mediated activation of the IKK complex. The K63/M1-linked hybrid polyubiquitin chains may contain recently identified branched chains [65].

Extensive studies on the role of Tax in ATL development have demonstrated that Tax is involved in leukemogenesis largely through its ability to constitutively activate NF-κB [7–9]. It has been known for almost two decades that the binding of Tax to NEMO is required for IKK activation, but the precise molecular mechanisms by which this binding leads to IKK activation remain to be elucidated. We and other groups have shown that the Tax-induced generation of K63 chains is crucial for IKK activation [27, 28, 30]. Furthermore, Ho et al. [28] recently provided clear evidence that RNF8 acts as an E3 ligase to generate K63 chains for IKK activation. As an extension of these previous results, we propose a novel molecular model for Tax-induced IKK activation in which LUBAC, together with unidentified E3 ligases for K63 chains, generate K63/M1-linked hybrid chains to form the active macromolecular Taxisome, composed of LUBAC, Tax and the active IKK complex, thereby establishing persistent NF-κB activation (Fig 7). Cell-free experiments allowed us to address the effects of chain type-specific blocking of polyubiquitin synthesis on critical steps of Tax-induced IKK activation. Several lines of evidence presented here support our model. 1) *In vitro* binding and immunoprecipitation experiments revealed that Tax can bind to both the IKK complex and LUBAC to form an inactive pre-Taxisome without the generation of polyubiquitin chains, whereas activation of the IKK complex by Tax requires the synthesis of the K27, K63 and M1 chains. 2) Genetic evidence revealed that both Ubc13 (an E2 enzyme for K63 chain synthesis) and each component of LUBAC (the only E3 enzyme for M1 chain) are crucial for Tax-induced IKK activation. 3) Mass spectrometric analyses revealed that both K63 and M1 chains are associated with the IKK complex only in the presence of Tax. 4) Cell-free experiments with chain type-specific DUBs revealed that K63/M1-linked hybrid chains are associated with the active IKK complex. 5) Tax-induced IKK activation requires the ability of NEMO to bind to both K63 and M1 chains. 6) The formation of the active macromolecular IKK complex (active Taxisome) requires the synthesis of the K27, K63 and M1 chains. Based on 4), 5) and 6), a single hybrid chain may bind to multiple NEMO molecules, and a single NEMO may act as a bridge between the hybrid chains, which may explain why generation of the hybrid chain is required for the macromolecular IKK complex. Regarding how the formation of the macromolecular complex leads to IKK activation, there are two potential mechanisms. The first is that the formation of the macromolecular complex could induce an efficient physical interaction between IKK complexes, so that *trans*-autophosphorylation between IKK complexes results in full activation of IKK. The second possible mechanism is that the formation of the macromolecular complex could somehow recruit an IKK kinase (IKKK) such as TAK1, which phosphorylates and activates IKK in response to cytokine stimulation [59]. We previously reported that MAP3Ks, including MEKK1, MEKK3, NIK, TPL-2 and TAK1, are dispensable for Tax-induced IKK activation [60]. In addition, our extensive proteomics analysis of the Tax-activated IKK complex failed to identify any IKKK candidates [61], leading us to prefer the *trans*-autophosphorylation model.

In contrast to our model, conflicting results have been reported on the following three points. The first point is the involvement of IKKK. Yin et al. [62] demonstrated that a dominant negative MEKK1 mutant inhibits IKK activation induced by Tax. Ho et al. [28] and Wu et al. [63] reported that Tax fails to activate the IKK complex in TAK1-knockdown HeLa cells or TAK1-knockout MEFs. This discrepancy may due to the experimental conditions including cell types used. Extensive analysis of the macromolecular complex and genetic and biochemical experiments in T cells are required to determine the involvement of any IKKK in Tax-induced IKK activation. The second point concerns the E3 ligase for K63 chain formation. Wang et al. [29] recently reported that Tax acts as an E3 ubiquitin ligase for IKK activation through synthesis of mixed-linkage polyubiquitin chains and that K63 chains are dispensable for Tax-induced IKK activation. However, we show that K63 chains are essential and could not demonstrate the E3 activity of Tax under any conditions we tested. Further identification of factors involved in Tax-induced IKK activation may explain these discrepancies. The third point concerns the subcellular localization of the active Taxisome. Pujari et al. [64] reported that membrane-associated Cell adhesion molecule 1 (CADM1) functions as a scaffold for Tax and Ubc13 to activate the IKK complex in intact cells. Since the S-100 fraction used in the cell-free system does not contain membrane fractions, we believe that Tax can interact with Ubc13 in the absence of CADM1. However, our data do not exclude the possibility that, in the cell free assay, Tax induces IKK activation without molecules required for subcellular localization in intact T cells. Precise comparison of IKK activation in the cell-free system with that in intact cells will explain regulation of Taxisome formation in HTLV-1 infected cells

Inconsistent with our model, the regular complex (dots in Fig 5F), which was inactive in the absence of Tax, appeared to be activated in the presence of Tax in addition to the macromolecular complex. This could occur because the macromolecular complex is unstable, such that the activated macromolecular complex dissociates into the active regular-size complex, or because the active macromolecular complex is able to transiently associate with and activate the regular complex. However, we cannot completely rule out the possibility that the IKK complex can be activated without the formation of the macromolecular complex in the presence of Tax.

Interestingly, cell-free experiments using IsoT and viral OTU revealed that both unanchored and anchored polyubiquitin chains are required for Tax-induced IKK activation. Although the critical roles of unanchored and anchored polyubiquitin chains in Tax-induced IKK activation are still controversial, our experiments using Tax KR mutants support the idea that the polyubiquitin chains conjugated to Tax belong to the class of substrate-anchored polyubiquitin chains required for Tax-induced IKK activation. By taking advantage of specific DUBs that degrade only one type, we showed for the first time that unanchored and anchored polyubiquitin chains cooperate in Tax-induced IKK activation.

Although the addition of the DN ubiquitin mutant K27R inhibited IKK activation by Tax, we did not detect K27 chains associated with the active Taxisome via the ubiquitin-AQUA method. This may be because an undetectable amount of K27 chains is involved in the generation of the hybrid chains as their component or because K27 chains act as initial triggers of the hybrid chains synthesis but are dissociated from the Taxisome afterwards. Further studies are needed to identify the precise role of K27 chains in Tax-induced IKK activation.

Tax binds to LUBAC through the UBL domain of HOIL-1L and RBR domain of HOIP. The UBL domain of HOIL-1L is involved in the association with HOIP, while the RBR domain of HOIP is the catalytic active site [66]. Therefore, the association of Tax with LUBAC may activate the E3 activity of LUBAC upon HTLV-1 infection, which may be one of the critical events in the onset of ATL. In accord with this scenario, small compounds that specifically block the interaction between Tax and HOIL-1L or between Tax and HOIP could be used as novel therapeutic approaches for ATL and HAM.

## Materials and Methods

### Plasmids

Human cDNAs encoding dominant-negative mutants of E2 enzymes were generated via PCR and inserted into the pRK5 vector. pMRX-Cre was obtained from S. Akira (Osaka University). Expression vectors for HOIL-1L-HA, Myc-HOIP and their deletion mutants were generated as previously described [66]. Human and mouse cDNAs encoding NEMO and NEMO mutants were inserted into the retrovirus vector pMXs obtained from T. Kitamura (University of Tokyo). Viral OTU cDNA was obtained from A. García-Sastre (Icahn School of Medicine at Mount Sinai). 3xκB-luc was obtained from S. Miyamoto (University of Wisconsin-Madison). Tax cDNA was obtained from J. Fujisawa (Kansai Medical University). Tax mutants were generated via PCR and inserted into the pCG vector.

### Antibodies

The following antibodies were used: anti-p-IκBα (9246), anti-IκBα (9242), anti-p-IKKα/β (2697), anti-NEMO (2695) and anti-ubiquitin (3936) (Cell Signaling Technology); anti-GST (sc-459), anti-Myc (sc-789) and anti-HA probe (sc-805) (Santa Cruz Biotechnology); anti-Flag M2 (F3165) (Sigma); anti-tubulin (CP06) (Calbiochem); anti-HOIL-1L (NBP-1-88301) (Novus Biologicals); anti-HOIP (ARP43241) (Aviva Systems Biology); anti-Sharpin (14626-1-AP) (Proteintech); anti-linear polyubiquitin-specific (AB130) (LifeSensors); and anti-Ubc13 (37–1100) (ThermoFisher Scientific). The anti-Tax antibody was generated as previously described [67].

### Cell culture and transfection

HEK293T cells (purchased from ATCC), Plat-E cells (provided by T. Kitamura) and mouse embryonic fibroblasts (provided by J. Silke, The Walter and Eliza Hall Institute, or established by us) were maintained in Dulbecco’s modified Eagle’s medium (DMEM) supplemented with 10% heat-inactivated fetal bovine serum (FBS). Jurkat cells (purchased from ATCC), the Jurkat-derived cell line JPX-9 cells (provided by K. Ohtani, Kwansei Gakuin University), and HTLV-1-infected cell line HUT102, MT-2, and MT-4 cells (provided by J. Fujisawa) were maintained in RPMI1640 supplemented with 10% heat-inactivated FBS. Sf9 cells were maintained in Sf900IIISFM (Thermo Fisher Scientific) supplemented with 10% FBS. The transfection of HEK293T cells was performed by the calcium phosphate method. siRNAs were transfected using NEPA21 Super Electroporator (NEPAGENE). Control stealth siRNA and the following stealth siRNAs (Thermo Fisher Scientific) were used: HOIP-1 sense/anti-sense, 5′-GGUACUGGCGUGGUGUCAAGUUUAA-3′/5′-UUAAACUUGACACCACGCCAGUACC-3′; HOIP-2 sense/anti-sense, 5′-CACCACCCUCGAGACUGCCUCUUCU-3′/5′-AGAAGAGGCAGUCUCGAGGGUGGUG-3′.

### Lentiviral infection

For lentivirus production, HEK293T cells were transfected with the self-inactivating lentiviral vector construct, the packaging construct and the VSV-G- and Rev-expressing construct. After 48 h of incubation, culture supernatants were collected and centrifuged at 50,000 x g for 1 h at 20°C to concentrate lentivirus. MT-2 or MT-4 cells were infected with the lentivirus at 400 x g for 2 h at 20°C. After 48 h, puromycin (Wako) was added to the medium, and puromycin-resistant cell pools were used for further experiments. The following target sequences were used: Tax-1, 5′-GGCCTTCCTCACCAATGTTCC-3′; Tax-2, 5′-GGCAGATGACAATGACCATGA-3′; Tax-3, 5′-GCCTACATCGTCACGCCCTAC-3′; HOIP-1, 5′-GCTGCAGCTTTCAGAATTTGA-3′; HOIP-2, 5′-GCACTGCCCATCCTGTAAACA-3′; HOIP-3, 5′-GCTCCTTTGGCTTCATATATG-3′; Control, 5′-GATTTCGAGTCGTCTTAATGT-3′.

### Retroviral infection

For retrovirus production, Plat-E cells were transfected with pMRX-Cre vector. After 24 h, culture supernatants were collected, and *Ubc13*^+/+^ or *Ubc13*^fl/fl^ MEFs were incubated with the retrovirus containing polybrene (10 μg/ml; Sigma-Aldrich) for 8 h. After 48 h, puromycin was added to the medium, and puromycin-resistant cell pools were used for further experiments.

### Recombinant proteins

His_6_-Tax, His_6_-M22, GST-viral OTU (WT) and its catalytic inactive mutant 1A were expressed in *E*. *coli* and purified. His_6_-TRAF6 and His_6_-Tax were expressed in Sf9 cells using the Bac-to-Bac Baculovirus Expression System (Thermo Fisher Scientific) and purified. GST, GST-HOIL-1L, GST-HOIP and GST-Sharpin were generated using the wheat germ cell-free protein synthesis system and purified [68]. Ubiquitin (U-100H), ubiquitin mutants (K6R (UM-K6R), K11R (UM-K11R), K27R (UM-K27R), K29R (UM-K29R), K33R (UM-K33R), K48R (UM-K48R) and K63R (UM-K63R)), HA-ubiquitin (U-110), IsoT (E-320), Otubain-1 (E-522B), AMSH (E-548B), OTULIN (E-558), USP2 (E-504), His_6_-UBE1 (E-304), UbcH5c (E2-627), UbcH7 (E2-640) and His_6_-Ubc13/Uev1A (E2-664) were purchased from BostonBiochem. Recombinant K48- and K63-linked Ub_2_-Ub_7_ chains (UC-230, UC-330) were purchased from BostonBiochem. Recombinant M1-linked Ub_2_-Ub_7_ chains (BML-UW1010-0100) were purchased from Enzo Life Sciences.

### Cell-free assays

Jurkat cells and MEFs were suspended in hypotonic buffer (10 mM Tris-HCl (pH 7.5), 1.5 mM MgCl_2_, 10 mM KCl, 0.5 mM dithiothreitol (DTT) and protease inhibitor cocktail (Roche)) and then lysed with a Dounce homogenizer. Cell debris was removed via ultracentrifugation at 100,000 x g for 1 h at 4°C to prepare the S-100 cytosolic extract. Cytosolic extracts (10 mg/ml) were incubated with recombinant His_6_-Tax in ATP buffer (50 mM Tris-HCl (pH 7.5), 5 mM MgCl_2_, 2 mM ATP, 5 mM NaF, 20 mM β-glycerophosphate, 1 mM Na_3_VO_4_, and protease inhibitor cocktail) in the presence or absence of various recombinant DUBs. After incubation at 30°C for 1 h, the reaction mixtures were subjected to immunoblotting or immunoprecipitation.

### Immunoprecipitation and immunoblotting

Cells were lysed in IP buffer (20 mM Tris-HCl (pH 7.5), 150 mM NaCl, 2 mM EDTA, 1 mM MgCl_2_, 10 mM NaF, 1% NP-40, 10 mM β-glycerophosphate, 1 mM Na_3_VO_4_, 1 mM DTT, 5 mM N-ethylmaleimide (NEM) and protease inhibitor cocktail), followed by centrifugation at 22,000 x g for 15 min at 4°C to remove the insoluble fraction. For detection of polyubiquitination of Tax, the reaction mixtures were boiled for 10 min in the presence of 1% SDS to remove noncovalently attached proteins. The mixtures were then diluted 10-fold in IP buffer to reduce the SDS concentration to 0.1%. The cell lysates or the cell-free reaction mixtures were subsequently incubated with the antibodies plus protein G-sepharose. The immunoprecipitates were washed five times and subjected to immunoblotting. For immunoblotting, immunoprecipitates or cell lysates were separated via SDS-PAGE and transferred to PVDF membranes (Immobilon P, Millipore). The membranes were then incubated with the primary antibodies. Immunoreactive proteins were visualized with anti-rabbit or anti-mouse IgG conjugated to horseradish peroxidase, followed by processing with an ECL detection system.

### *In vitro* binding assay

Glutathione sepharose was incubated with 300 ng of GST, GST-HOIL-1L, GST-HOIP or GST-Sharpin at 4°C for 1 h in binding buffer (50 mM Tris-HCl (pH 7.5), 150 mM NaCl, 1 mM EDTA, 1% Triton X-100, 1 mM DTT, 2.5 mg/ml BSA and protease inhibitor cocktail). The beads were then incubated with 500 ng of His_6_-Tax at 4°C for 1 h. After incubation, the beads were washed and subjected to immunoblotting.

### *In vitro* deubiquitination assay

Jurkat cytosolic extracts (10 mg/ml) were incubated with recombinant His_6_-Tax in ATP buffer. After incubation at 30°C for 1 h, the reaction mixtures were subjected to immunoprecipitation with an anti-NEMO antibody in IP buffer. The immunoprecipitates were washed three times with IP buffer without NEM and incubated with Otubain-1 (5 μM), AMSH (5 μM), OTULIN (5 μM) or USP2 (5 μM) at 37°C for 1 h in DUB buffer (50 mM HEPES-KOH (pH 7.5), 100 mM NaCl, 1 mM MnCl_2_, 0.01% Brij-35 and 2 mM DTT). After incubation, the reaction mixtures were subjected to immunoblotting.

### Ubiquitin-AQUA

Jurkat cytosolic extracts (10 mg/ml) were incubated with recombinant His_6_-Tax in ATP buffer. After incubation at 30°C for 1 h, the reaction mixtures were subjected to immunoprecipitation with an anti-NEMO antibody in IP buffer. The immunoprecipitates were analyzed via the ubiquitin-AQUA method as described previously [48]. The immunoprecipitates were separated through SDS-PAGE, and the gel region above 50 kDa was subjected to in-gel trypsinization. The extracted peptides were analyzed with a Q Exactive mass spectrometer in targeted MS/MS mode together with 10 fmol of ubiquitin AQUA peptides.

### Blue native polyacrylamide gel electrophoresis (BN-PAGE)

After the cell-free reaction, the reaction mixtures were mixed with NativePAGE Sample Buffer (Thermo Fisher Scientific). Electrophoresis was performed using NativePAGE Running Buffer (Thermo Fisher Scientific) containing 0.002% G-250. The gels were soaked in denaturation buffer (10 mM Tris-HCl (pH 6.8), 1% SDS and 0.006% 2-mercaptoethanol) for 30 min at 60°C, followed by transfer to PVDF membranes and immunoblotting.

### Quantitative real-time reverse transcriptase PCR

The total RNA was isolated from MT-4 cells transfected with control or HOIP siRNA with Trizol reagent (Thermo Fisher Scientific). cDNA was synthesized from 2.0 μg of total RNA with Prime ScriptII (Takara). Quantitative real-time PCR analysis was performed on CFX Connect (Bio-Rad). The level of GAPDH expression was used to normalize the data. The following primers were used: IL-6 sense/anti-sense, 5′-CCTGAACCTTCCAAAGATGGC-3′/5′-TTCACCAGGCAAGTCTCCTCA-3′; IL-1β sense/anti-sense, 5′-TTCGACACATGGGATAACGAGG-3′/5′-TTTTTGCTGTGAGTCCCGGAG-3′; MMP-9 sense/anti-sense, 5′-ATGTACCGCTTCACTGAGGG-3′/5′-TCAGGGCGAGGACCATAGAG-3′; GAPDH sense/anti-sense, 5′-TGCACCACCAACTGCTTAGC-3′/5′-GGCATGGACTGTGGTCATGAG-3′.

### Luciferase assay

HEK293T cells were transfected with the plasmids encoding wild type or various Tax mutants together with 20 ng of luciferase reporter (3xκB-luc) and 30 ng of β-actin-β-galactosidase plasmid. After 48 h, the luciferase activity was measured using the Luciferase Assay System (Toyo Ink). β-galactosidase activity was used to normalize the transfection efficiency.

### *In vitro* ubiquitination assay

The reactions were performed at 37°C for 1 h in the reaction buffer containing His_6_-UBE1 (0.1 μM), the indicated E2 (0.2 μM), ubiquitin (25 μM) and His_6_-TRAF6 or His_6_-Tax in the presence of ATP (2 mM). After incubation, the reaction mixtures were analyzed by immunoblotting.

### Phos-tag SDS-PAGE

After cell-free reactions, the reaction mixtures were separated using 6% polyacrylamide gels containing 20 μM Phos-tag acrylamide (Wako) and 40 μM MnCl_2_. After electrophoresis, the gels were washed with transfer buffer containing 10 mM EDTA for 15 min. The gels were further washed with transfer buffer without EDTA for 10 min, and the samples were transferred to PVDF membranes, followed by immunoblotting.

### Statistics

Statistically significant differences between mean values were determined using Student’s *t*-test (**P<0.01, *P<0.05). Data are presented as the means ± SD.

## Supporting Information

S1 Fig Proteasomal degradation of IκBα is not induced in cell-free assay system. Jurkat cytosolic extracts were incubated with recombinant His_6_-Tax and ATP (2 mM) in the presence of MG132 (10 μM). The reaction mixtures were analyzed by immunoblotting with the indicated antibodies.
(TIF)

S2 Fig cIAP1/2, TRAF2/5, TRIM25 and Riplet are dispensable for Tax-induced IKK activation. **(A-D)** Cytosolic extracts were prepared from cIAP1/cIAP2-deficient **(A)**, TRAF2/TRAF5-deficient **(B)**, TRIM25-deficient **(C)**, Riplet-deficient MEFs **(D)** and corresponding WT MEFs and subjected to cell-free analyses. The depicted results are representative of three independent experiments.
(TIF)

S3 Fig The zinc finger of Tax is crucial for IKK activation, while Tax does not act as an E3 ubiquitin ligase to generate K63 chains. **(A)** A schematic representation of the various Tax mutants used in **(B)** and **(C)**. **(B)** HEK293T cells were transfected with expression plasmids encoding Tax or various Tax mutants. After 60 h, the cells were treated with MG132 (20 μM) for 2 h, and the cell lysates were subjected to immunoblotting with the indicated antibodies. **(C)** HEK293T cells were transfected with plasmids encoding Tax or various Tax mutants together with a 3xκB-luc reporter. After 48 h, luciferase activity was measured. The results are given as the mean ±S.D. (n = 3). **(D)** Jurkat cytosolic extracts were incubated with recombinant His_6_-Tax purified from Sf9 cells or *E*. *coli* in the presence of ATP (2 mM). The reaction mixtures were analyzed by immunoblotting with the indicated antibodies. His_6_-Tax from Sf9 is larger than that from *E*. *coli* due to the difference in the length of linker sequence between His-tag and Tax protein. **(E)** Recombinant His_6_-Tax purified from Sf9 cells or *E*. *coli* (left) or His_6_-TRAF6 (right) was incubated with UBE1 (E1; 0.1 μM), the indicated E2 (0.2 μM) and ubiquitin (25 μM) in the presence of ATP (2mM). The reaction mixtures were analyzed by immunoblotting with an anti-Ub antibody. The depicted results are representative of three independent experiments.
(TIF)

S4 Fig HOIP becomes phosphorylated by IKKβ during Tax-induced IKK activation. **(A)** Jurkat cytosolic extracts were incubated with recombinant His_6_-Tax and ATP (2 mM) in the presence of DN ubiquitin mutants or HA-ubiquitin (50 μM). The reaction mixtures were separated via Phos-tag SDS-PAGE, followed by immunoblotting with an anti-HOIP antibody. **(B)** Jurkat cytosolic extracts were incubated with recombinant His_6_-Tax and ATP (2 mM) in the absence or presence of increasing amounts of the IKKβ inhibitor TPCA-1 (1.0, 3.0 or 10 μM). The reaction mixtures were separated via regular SDS-PAGE. Dots denote the phosphorylated form of HOIP. The depicted results are representative of three independent experiments.
(TIF)
