# Supplementary figures and images for: HTLV-1 Tax Induces Formation of the Active Macromolecular IKK Complex by Generating Lys63- and Met1-Linked Hybrid Polyubiquitin Chains
Source: PLoS Pathog. 2017 Jan 19;13(1):e1006162. doi: 10.1371/journal.ppat.1006162 (PMC5283754; doi:10.1371/journal.ppat.1006162)

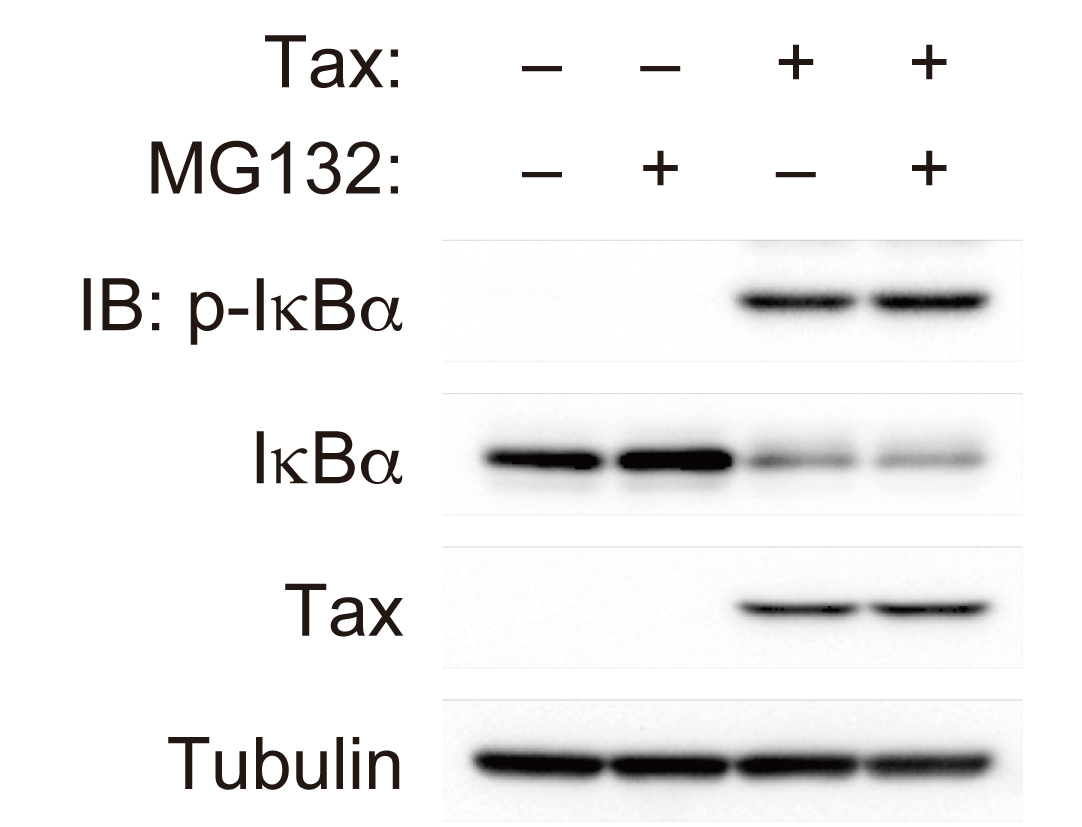

Supplement: S1 Fig — Jurkat cytosolic extracts were incubated with recombinant His6-Tax and ATP (2 mM) in the presence of MG132 (10 μM). The reaction mixtures were analyzed by immunoblotting with the indicated antibodies. (TIF) [file ppat.1006162.s001.tif]

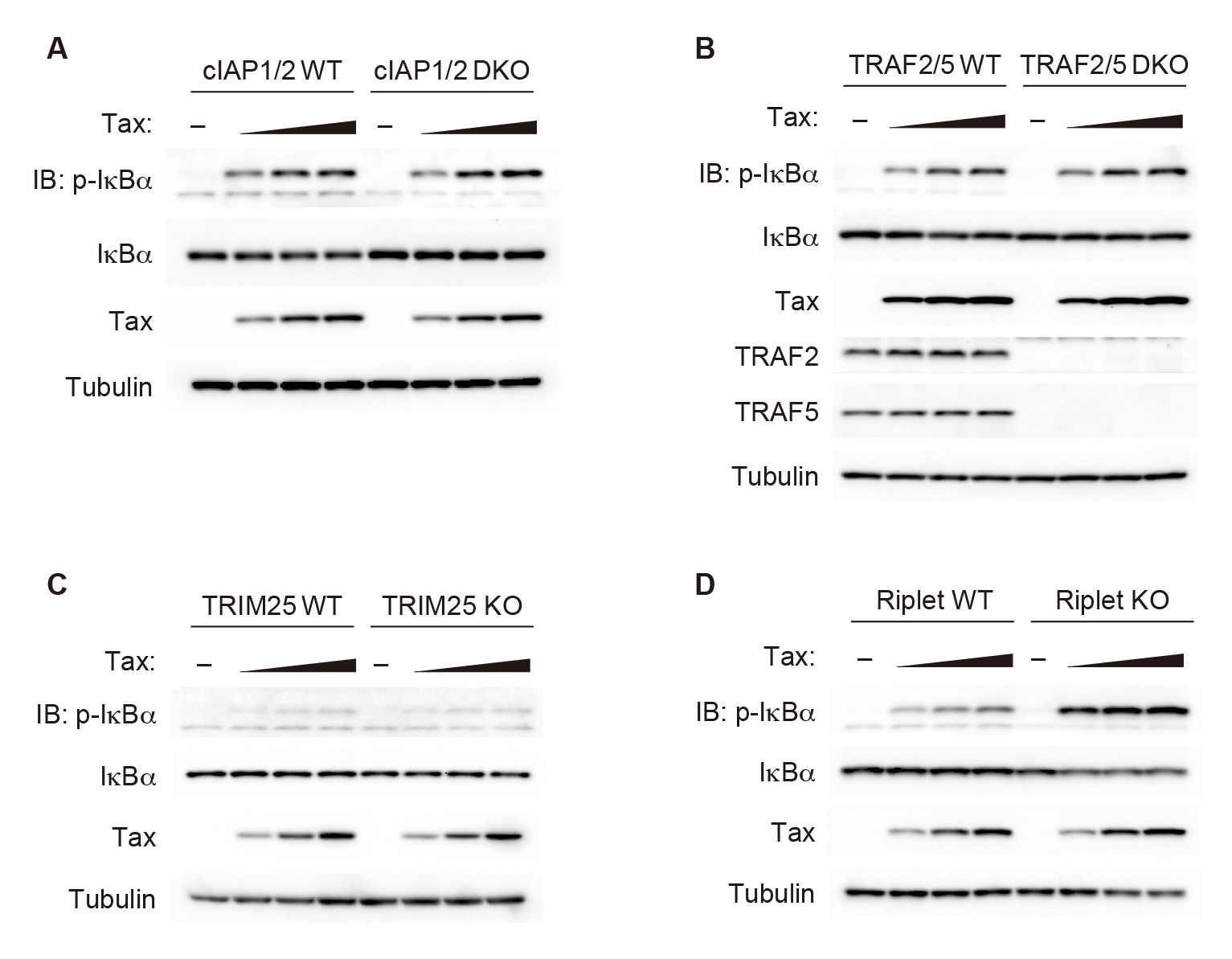

Supplement: S2 Fig — (A-D) Cytosolic extracts were prepared from cIAP1/cIAP2-deficient (A), TRAF2/TRAF5-deficient (B), TRIM25-deficient (C), Riplet-deficient MEFs (D) and corresponding WT MEFs and subjected to cell-free analyses. The depicted results are representative of three independent experiments. (TIF) [file ppat.1006162.s002.tif]

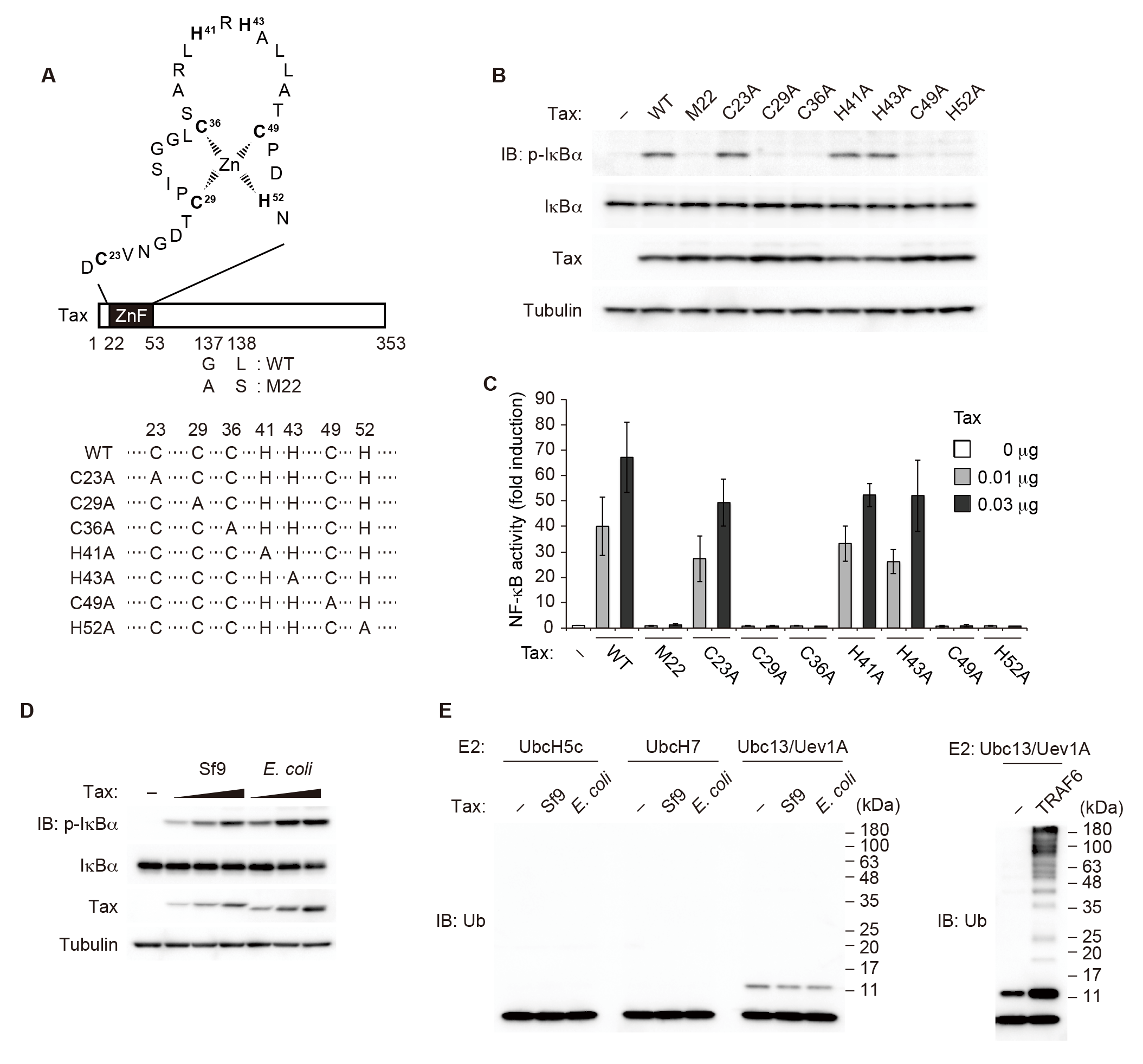

Supplement: S3 Fig — (A) A schematic representation of the various Tax mutants used in (B) and (C). (B) HEK293T cells were transfected with expression plasmids encoding Tax or various Tax mutants. After 60 h, the cells were treated with MG132 (20 μM) for 2 h, and the cell lysates were subjected to immunoblotting with the indicated antibodies. (C) HEK293T cells were transfected with plasmids encoding Tax or various Tax mutants together with a 3xκB-luc reporter. After 48 h, luciferase activity was measured. The results are given as the mean ±S.D. (n = 3). (D) Jurkat cytosolic extracts were incubated with recombinant His6-Tax purified from Sf9 cells or E. coli in the presence of ATP (2 mM). The reaction mixtures were analyzed by immunoblotting with the indicated antibodies. His6-Tax from Sf9 is larger than that from E. coli due to the difference in the length of linker sequence between His-tag and Tax protein. (E) Recombinant His6-Tax purified from Sf9 cells or E. coli (left) or His6-TRAF6 (right) was incubated with UBE1 (E1; 0.1 μM), the indicated E2 (0.2 μM) and ubiquitin (25 μM) in the presence of ATP (2mM). The reaction mixtures were analyzed by immunoblotting with an anti-Ub antibody. The depicted results are representative of three independent experiments. (TIF) [file ppat.1006162.s003.tif]

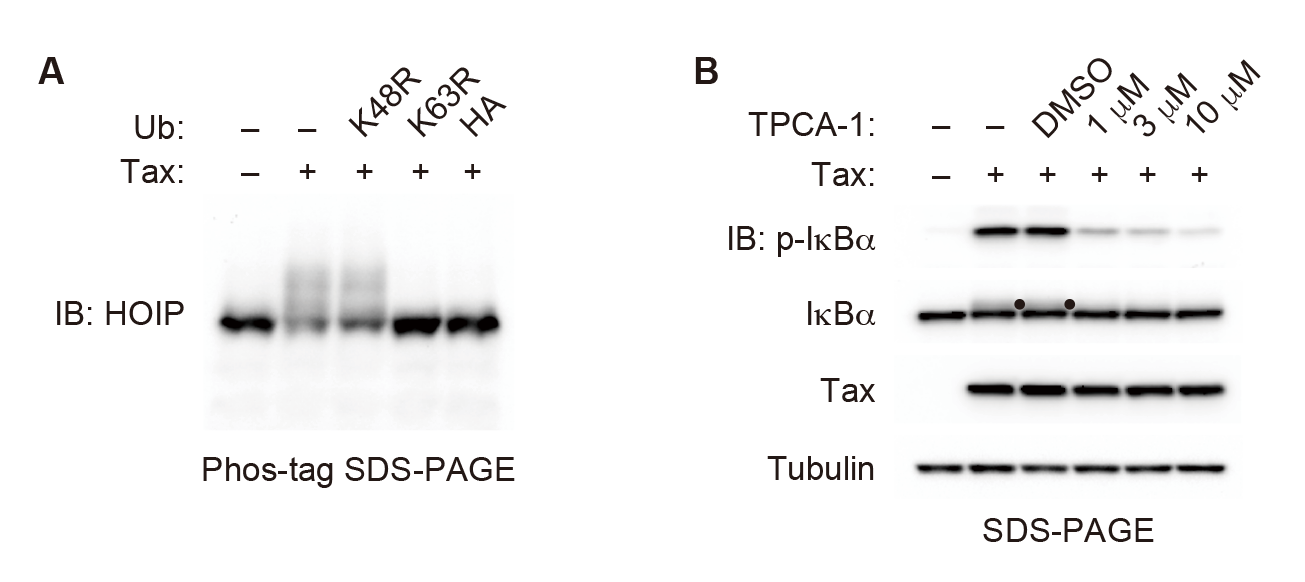

Supplement: S4 Fig — (A) Jurkat cytosolic extracts were incubated with recombinant His6-Tax and ATP (2 mM) in the presence of DN ubiquitin mutants or HA-ubiquitin (50 μM). The reaction mixtures were separated via Phos-tag SDS-PAGE, followed by immunoblotting with an anti-HOIP antibody. (B) Jurkat cytosolic extracts were incubated with recombinant His6-Tax and ATP (2 mM) in the absence or presence of increasing amounts of the IKKβ inhibitor TPCA-1 (1.0, 3.0 or 10 μM). The reaction mixtures were separated via regular SDS-PAGE. Dots denote the phosphorylated form of HOIP. The depicted results are representative of three independent experiments. (TIF) [file ppat.1006162.s004.tif]
